# The Role of Urinary Nitrite in Predicting Bacterial Resistance in Urine Culture Analysis Among Patients With Uncomplicated Urinary Tract Infection

**DOI:** 10.7759/cureus.26032

**Published:** 2022-06-17

**Authors:** Vladimer Papava, Tamar Didbaridze, Zurabi Zaalishvili, Nino Gogokhia, Giorgi Maziashvili

**Affiliations:** 1 Urology, Tbilisi State Medical University, Tbilisi, GEO; 2 Microbiology, Tbilisi State Medical University, Tbilisi, GEO; 3 Clinical Microbiology, Tbilisi State Medical University the First University Clinic, Tbilisi, GEO; 4 Medicine, Faculty of Medicine, Tbilisi State Medical University, Tbilisi, GEO; 5 Laboratory Medicine, Tbilisi State Medical University the First University Clinic, Tbilisi, GEO

**Keywords:** antibiotic resistance, anti-bacterial agents, escherichia coli, nitrite, dysuria, urinary tract infection

## Abstract

Objective

This study aims to determine the relationship between the presence of urinary nitrite and bacterial resistance to antimicrobial therapy in patients with uncomplicated urinary tract infections.

Methods

During a six-month time period (April-October, 2020), we reviewed the urine samples of 59 adult outpatients from the Urology Department of Tbilisi State Medical University the First University Clinic with the diagnosis of urinary tract infection. The infecting microorganisms and the presence of urine nitrite were recorded. Resistance rates to the antibiotics were compared between the positive and negative nitrite groups. Chi-squared test was used to perform the statistical analysis using Prism software version 9.3.1 (GraphPad Software, Inc., San Diego, California).

Results

We examined the correlation between the nitrite-positive and -negative groups with the resistance pattern to ceftriaxone, trimethoprim/sulfamethoxazole (TMP-SMX), ampicillin-sulbactam, fosfomycin, amikacin, doxycycline, cefuroxime, cefotaxime, ceftazidime, and nitrofurantoin.

A total of 59 outpatients with a mean age of 37 years met the inclusion criteria between April and October 2020. In the positive and negative nitrite groups, there were 23 and 36 patients, respectively. Three (17.6%) of the 17 gram-positive organisms and 20 (62.5%) of the 42 gram-negative organisms yielded positive nitrite results.

In nitrite-positive group, resistance rates to ceftriaxone, TMP-SMX, ampicillin-sulbactam, fosfomycin, amikacin, doxycycline, cefuroxime, cefotaxime, ceftazidime, and nitrofurantoin were 52.2%, 70.8%, 63.5%, 67.7%, 25.8%, 31.9%, 29.6%, 32.5%, 22.5% and 83.8%, respectively. These values in the nitrite-negative group were 6.5%, 41.3%, 60.7%, 72.9%, 49%, 3%, 2.3%, 3.3%, 4.3% and 81.9%, respectively.

Highest relative resistance rate was recorded against cefuroxime (12.9), followed by doxycycline (10.6), cefotaxime (9.8), ceftriaxone (8.03), ceftazidime (5.2), TMP-SMX (1.71), ampicillin-sulbactam (1.05), nitrofurantoin (1.02), fosfomycin (0.93), and amikacin (0.53).

The most commonly isolated pathogen was *Escherichia coli*, which was detected in 35 (71%) isolates. Other bacteria commonly found were *Proteus *spp in five (12%) isolates, *Klebsiella* spp in two (5%) isolates, and *Enterococcus *in five (12%) isolates.

Conclusion

The findings revealed that out of 10 antibiotics, nitrite-positive groups demonstrated higher resistance only against ceftriaxone, cefuroxime, cefotaxime, and doxycycline. Other antibiotics showed no statistically significant differences in resistance. Furthermore, the highest relative resistance rate was recorded against cefuroxime, whereas amikacin revealed the lowest. Therefore, we suggest physicians to not adjust antibiotic therapy for urinary tract infections (UTIs) based on the presence of nitrite. Urine bacteriology should be ordered.

## Introduction

Urinary tract infection (UTI) is a common disease. It is caused by pathogenic microorganisms (bacteria, viruses, or fungi) invading the urinary tract system [1,2]. Cystitis, pyelonephritis, and asymptomatic bacteriuria are the three manifestations of the condition [1,2]. In 70%-90% of cases, *Escherichia coli* is the etiologic agent [1,2]. Early detection and timely and adequate treatment of the disease are critical to preventing major complications such as renal scarring, hypertension, and chronic renal failure [3,4]. Regarding the rising treatment resistance of microorganisms causing UTIs, appropriate selection of the initial antibiotic before collecting urine culture and antibiogram results are critical [1,2,5-9]. The question is whether it is possible to predict microorganism resistance using markers, such as the nitrite test, prior to preparing urine cultures and antibiogram results. According to Weisz et al., urine nitrite results can be used to identify cephalosporin resistance [10]. On the other hand, other studies contradict this viewpoint [11,12].

Antimicrobial resistance is increasing globally, making infections more difficult to treat, and is related to increased mortality, morbidity, and cost [13,14]. A growing proportion of community-acquired uncomplicated UTIs is caused by multidrug-resistant gram-negative bacilli. As a result, empirical therapy is more likely to fail. Because there are no oral treatment alternatives, an increasing number of individuals with uncomplicated UTIs require hospitalization for intravenous antibiotics.

Nitrite tests look for the metabolites of nitrite reductase, an enzyme generated by a variety of microorganisms. Unless there is a UTI, these compounds are not generally present. This test has a sensitivity and specificity of 25% and 94%-100%, respectively. The limited sensitivity has been linked to infection caused by enzyme-deficient bacteria or low-grade bacteriuria. A positive nitrite test result is fairly specific for UTI, mainly due to urease-positive organisms such as *Proteus *species and, on rare occasions, *E. coli*; nevertheless, it is very insensitive as a screening tool, with only 25% of UTI patients having a positive nitrite test result.

The link between urinary nitrite and UTIs was initially documented in 1914 and has since been the subject of extensive attention [2]. The benefits of using urinary nitrites include the low cost, the speed with which the results are provided, and the ability to categorize patients into two distinct groups - nitrite positive or negative [14].

Knowing the pathogen spectrum and resistance patterns in the community allows the physician to choose an effective empiric agent. There has been little research on whether the absence of urine nitrites reflects resistance to popular antibiotics used to treat uncomplicated UTIs. Furthermore, the findings of the few studies that have looked into this correlation are contradictory [15]. This study aimed to investigate if nitrite findings in urinalysis could be utilized to guide bacterial isolate and resistance when treating UTIs.

## Materials and methods

The urine samples of 59 adult outpatients with a mean age of 37 years and a diagnosis of UTI were reviewed. The Urology Department at Tbilisi State Medical University (TSMU) the First University Clinic provided samples throughout a six-month period (April-October 2020). Patients who were below the age of 18 years, who did not have a urine culture sent to the laboratory, or those with the diagnosis of UTI but a negative culture were excluded from the study.

The tests for bacterial identification and antibiotic sensitivity were carried out at the clinical microbiology laboratory of TSMU the First University Clinic. Samples were promptly transported to the laboratory and processed within 30 minutes of being collected. If a delay of more than one to two hours was expected, the specimen was refrigerated. All specimens were cultured on blood agar and MacConkey agar for 24 hours at 37°C.

All samples were subjected to the following tests: (a) gram staining of the colonies, (b) biochemical reactions using the Analytical Profile Index (API) identification system (API20E, API20 NE, APIstaph, APIstrep; bioMérieux, France), and (c) identification and antimicrobial sensitivity testing using the Kirby-Bauer disk diffusion method.

Ceftriaxone, trimethoprim-sulfamethoxazole (TMP-SMX), ampicillin-sulbactam, fosfomycin, amikacin, doxycycline, cefuroxime, cefotaxime, ceftazidime, and nitrofurantoin were all tested on isolated bacteria. The European Committee on Antimicrobial Susceptibility Testing (EUCAST) standard was used to assess antibiotic susceptibility/resistance. 

Statistical analysis was performed using Prism software version 9.3.1 (GraphPad Software, Inc., San Diego, California). Chi-squared test was used to compare the variables. P-value ≤ 0.05 was considered statistically significant.

## Results

We examined the correlation between the nitrite-positive and -negative groups with the resistance pattern to ceftriaxone, TMP-SMX, ampicillin-sulbactam, fosfomycin, amikacin, doxycycline, cefuroxime, cefotaxime, ceftazidime, and nitrofurantoin. A total of 59 outpatients with a mean age of 37 years met the inclusion criteria between April and October 2020. Table 1 reveals that in the positive and negative nitrite groups, there were 23 and 36 patients, respectively. Three (17.6%) of the 17 gram-positive organisms and 20 (62.5%) of the 42 gram-negative organisms yielded positive nitrite results.

**Table 1:** Nitrite test results in gram-positive and gram-negative isolates

Gram stain/Nitrite test	Nitrite-positive	Nitrite-negative	Total
Gram-positive	3	14	17
Gram-negative	20	22	42
Total	23	36	59

**Figure 1:**
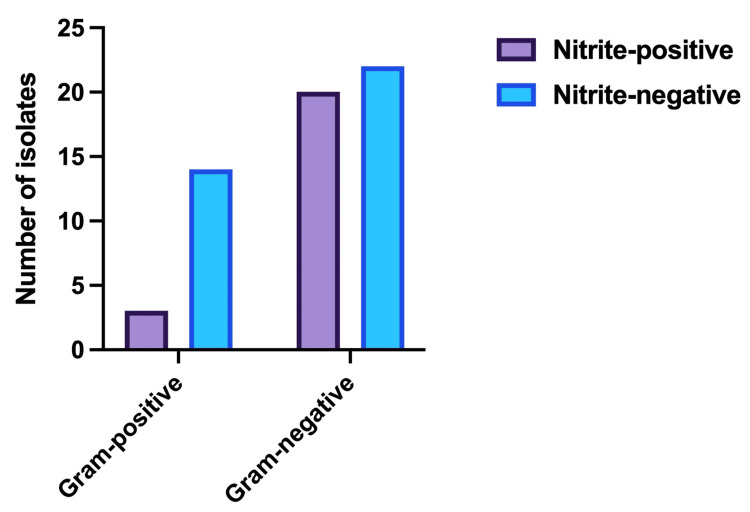
Gram stain and nitrite test pattern for isolated pathogens

The gram stain and nitrite test patterns for all isolated pathogens are shown in Figure 1.

Table 2 shows that in nitrite-positive group, resistance rates to ceftriaxone, TMP-SMX, ampicillin-sulbactam, fosfomycin, amikacin, doxycycline, cefuroxime, cefotaxime, ceftazidime, and nitrofurantoin were 52.2%, 70.8%, 63.5%, 67.7%, 25.8%, 31.9%, 29.6%, 32.5%, 22.5%, and 83.8%, respectively. These values in the nitrite-negative group were 6.5%, 41.3%, 60.7%, 72.9%, 49%, 3%, 2.3%, 3.3%, 4.3%, and 81.9%, respectively. Only ceftriaxone, cefuroxime, cefotaxime, and doxycycline showed a statistically significant difference (p < 0.05). Highest relative resistance rate was recorded against cefuroxime (12.9), followed by doxycycline (10.6), cefotaxime (9.8), ceftriaxone (8.03), ceftazidime (5.2), TMP-SMX (1.71), ampicillin-sulbactam (1.05), nitrofurantoin (1.02), fosfomycin (0.93), and amikacin (0.53).

**Table 2:** Antibiotic resistance rates in nitrite-negative and nitrite-positive groups TMP-SMX: Trimethoprim-sulfamethoxazole.

Antibiotics	Resistance rate in the nitrite-positive group (%)	Resistance rate in the nitrite-negative group (%)	P-value	Relative resistance rate in nitrite-positive versus nitrite-negative group
Ceftriaxone	52.2	6.5	0.00005	8.03
TMP-SMX	70.8	41.3	0.56	1.71
Ampicillin-sulbactam	63.5	60.7	1	1.05
Fosfomycin	67.7	72.9	1	0.93
Amikacin	25.8	49	0.62	0.53
Doxycycline	31.9	3	0.004	10.6
Cefuroxime	29.6	2.3	0.005	12.9
Cefotaxime	32.5	3.3	0.005	9.8
Ceftazidime	22.5	4.3	0.19	5.2
Nitrofurantoin	83.8	81.9	1	1.02

**Figure 2:**
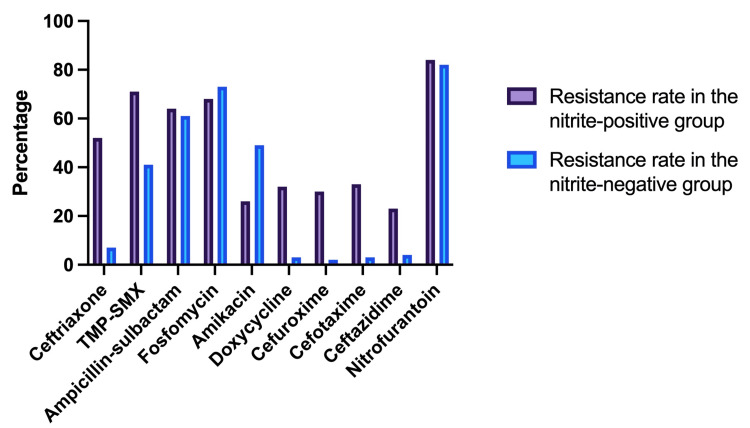
Comparison of resistance rates between the nitrite-positive and nitrite-negative groups TMP-SMX: Trimethoprim-sulfamethoxazole.

Figure 2 shows the correlation between nitrite findings and resistance patterns to various antibiotics that are being used to treat UTIs.

As depicted in Table 3, the most commonly isolated pathogen was *E. coli*, which was detected in 35 (71%) isolates. Other bacteria included *Proteus *spp in five (12%) isolates, *Klebsiella* spp in two (5%) isolates, and *Enterococcus *in five (12%) isolates.

**Table 3:** Frequency of isolated uropathogens

Pathogen	Number of isolates	Percentage of isolates (%)
Escherichia coli	35	71
*Proteus *spp	5	12
*Klebsiella *spp	2	5
Enterococcus	5	12

## Discussion

The purpose of this study was to see if the presence of urine nitrite could help clinicians choose the most appropriate empirical antibiotic before the bacteriological analysis findings. Commonly seen pathogens in complicated UTIs which do not convert nitrates to nitrites include *Enterococcus*, *Pseudomonas*, and *Acinetobacter *[16].

Weisz et al. stated that urine nitrite results are a useful sign of resistance to empiric therapy with first- and third-generation cephalosporins [10]. In our study, we subjected nitrite-positive and nitrite-negative groups to second and third-generation cephalosporins including cefuroxime, cefotaxime, ceftriaxone, and ceftazidime. In our study, only cefuroxime, cefotaxime, and ceftriaxone revealed a statistically significant difference (p ≤ 0.05) among these four antibiotics.

Larson et al. compared the sensitivity of nitrite-positive and nitrite-negative cultures to TMP-SMX. Results revealed that out of 86 nitrite-positive cultures, 22% showed resistance, whereas out of 73 nitrite-negative cultures, only 18% were resistant to the drug. There was no statistically significant difference in the proportion of TMP-SMX-resistant isolates [12]. Our findings revealed a similar pattern as we did not find a statistically significant difference between the two groups. Our results revealed the resistance rates of 70.8% and 41.3% in nitrite-positive and nitrite-negative groups, respectively. The variation in percentages may have been caused by the number of individual microorganisms included in the study since it is known that *Enterococci *tend to be more susceptible to TMP-SMX therapy [17].

Enterococcus, a less common uropathogen, does not produce nitrite and has a unique treatment resistance pattern [18]. Because of the expression of low-affinity penicillin-binding proteins, all enterococci have decreased sensitivity to penicillin and ampicillin but high-level resistance to most cephalosporins and all semi-synthetic penicillins [19].

A urine pH below 6.0, the amount of bacteriuria, the short time between collection and testing, dilute urine, and the presence of blood, urobilinogen, vitamin C, or medications can all cause a false-negative nitrite result. In addition, although several uropathogens, including *E. coli*, *Klebsiella*, and *Proteus*, can convert nitrate to nitrite, others cannot. Therefore, the nitrite test cannot be used to detect the presence of bacterial infection [10].

The main limitation of our study was the sampling size. Despite the fact that we found a statistically significant difference in resistance to four antibiotics, additional data is needed to draw a more certain conclusion.

## Conclusions

The findings revealed that out of 10 antibiotics, nitrite-positive groups demonstrated higher resistance only against ceftriaxone, cefuroxime, cefotaxime, and doxycycline. Other antibiotics showed no statistically significant differences in resistance. The highest relative resistance rate was recorded against cefuroxime, whereas amikacin demonstrated the lowest.

Based on our findings, we suggest physicians to not adjust antibiotic therapy for UTIs based on the presence of nitrite, and urine bacteriology should be ordered.
